# The influence of the chosen in vitro bone simulation model on intraosseous temperatures and drilling times

**DOI:** 10.1038/s41598-019-48416-6

**Published:** 2019-08-14

**Authors:** József Szalma, Bálint V. Lovász, László Vajta, Balázs Soós, Edina Lempel, Stephan C. Möhlhenrich

**Affiliations:** 1 University of Pécs, Medical School, Department of Oral and Maxillofacial Surgery, 5. Dischka, 7621 Pécs, Hungary; 2 University of Pécs, Medical School, Department of Conservative Dentistry and Periodontology, 5 Dischka, 7621 Pécs, Hungary; 3 Department of Orthodontics, University Hospital of Aachen University, Aachen, Germany; 4 Department of Oral and Maxillofacial Surgery, University Hospital of Aachen University, 30 Pauwelsstraße, 52074 Aachen, Germany

**Keywords:** Experimental models of disease, Preclinical research

## Abstract

There is no consensus about the most suitable *in vitro* simulating material investigating heat generation during bone preparation. The aim was to compare heat increases and drilling times of bone removals in different bone simulating materials and to compare them to fresh human cadaver bone. A cavity was drilled in the following samples: (1) bovine rib; (2) pig rib; (3) 20 PCF (lb/ft^3^) polyurethane (PU) block with 3 mm (50 PCF) cortical layer; (4) 20PCF PU without cortical; (5) 30 PCF PU with 2 mm (40 PCF) cortical; (6) 30 PCF PU with 1 mm (40 PCF) cortical; (7) 30PCF PU without cortical; (8) poly-methyl-methacrylate (PMMA); (9) fresh human cadaver rib. Data were analyzed with ANOVA followed by Tukey’s post hoc tests. P < 0.05 was considered significant. Highest heat increases and slowest drilling times were found in bovine ribs (p < 0.001). Regarding temperatures, human ribs were comparable to the pig rib and to PUs having cortical layers. Considering drilling times, the human rib was only comparable to the 20 PCF PU with 3 mm cortical and to 30 PCF PU without cortical. By the tested *in vitro* bone removals, only the 20 PCF PU with 3 mm cortical was able to simulate human ribs, considering both temperature increases and drilling times.

## Introduction

A significant part of surgical, implantology, orthopedic-traumatology and neurology literature address harmful intraosseous temperatures during bone removals and drilling maneuvers with the aim to reduce excessive heats in and in the near of bony tissues.

Drilling is a very complex machining process, cumulating the effects of cutting and material extrusion^1^. The heat generated is a result of friction between the drill bit and the cutting surface of the drilled hole and bone chips and fragments^2^. The produced heat is influenced significantly by *drill parameters* (diameter, material and design of the drill including cutting face, flutes, helices, drill point) and *drilling parameters* (drilling speed, axial load, feeding rate, predrilling, drilling depth, method of cooling, gradual drilling etc.)^3^. Bone density and the quality of the bone has a significant influence on intraosseous heat production, and very important differences exist whether the cortical or spongiform bone is drilled^3,4^. According to Möhlhenrich *et al*., the increasing artificial bone density correlated strongly with a temperature rise^5,6^.

According to a literature scan, the usually accepted thermal threshold, when thermal osteonecrosis occur is 47 °C lasting for one minute^7^, however, by higher temperatures much shorter time is enough to develop bone necrosis (e.g. 50 °C for 30 sec^8^ or 70–90 °C for few seconds^9,10^). Bone necrosis is the result of intracellular enzymatic and membrane protein denaturation, cell desiccation and dehydration, followed by membrane rupture and finally carbonization^4^. Thermal osteonecrosis may cause compromised wound healing after surgical tooth removals and in case of implant insertions it can impair osseointegration^3^.

For temperature measurements during drilling in the bone usually two basic methods are used. Thermocouple probes and the infrared thermography. Thermocouples allow direct temperature measurements inside the bone, but they detect only a single temperature point and need preparing pilot holes. Infrared thermography detects an overall thermal profile including also the measurement of leaking heat; however, it detects only the surface temperature, without predicting the temperature of the actual drilling site^1,2,6,11^. According to Möhlhenrich *et al*.’s systematic review, 18 studies used thermocouples, and 7 used thermal cameras of the involved, analyzed studies^12^.

Since, there is no universally recommended *in vitro* model, and researches use plenty different models, the comparison of different study results are almost impossible^12,13^. Moreover, there is very limited information, on how the choice of bone model influences outcomes by similar bone removals^13^. As Fernandes *et al*. (2017) wrote, to date just a relatively few numbers of papers have directly compared heat development of drilling in biological tissues with synthetic bones^1^. According to our best knowledge, this is the first attempt to compare porcine and bovine *ex vivo* models simultaneously with different density polyurethane blocks and with fresh human cadaver bone by the same bone drilling procedure (common in oral surgery bone removals^3^).

The purpose of this *in vitro* study was, to investigate a standardized bone drilling’s thermal effect and the duration of these preparations in different, frequently used bone simulating materials and in human bone to estimate the effect of the chosen model on the results.

## Materials and Methods

### Experimental set-up

**Figure 1:**
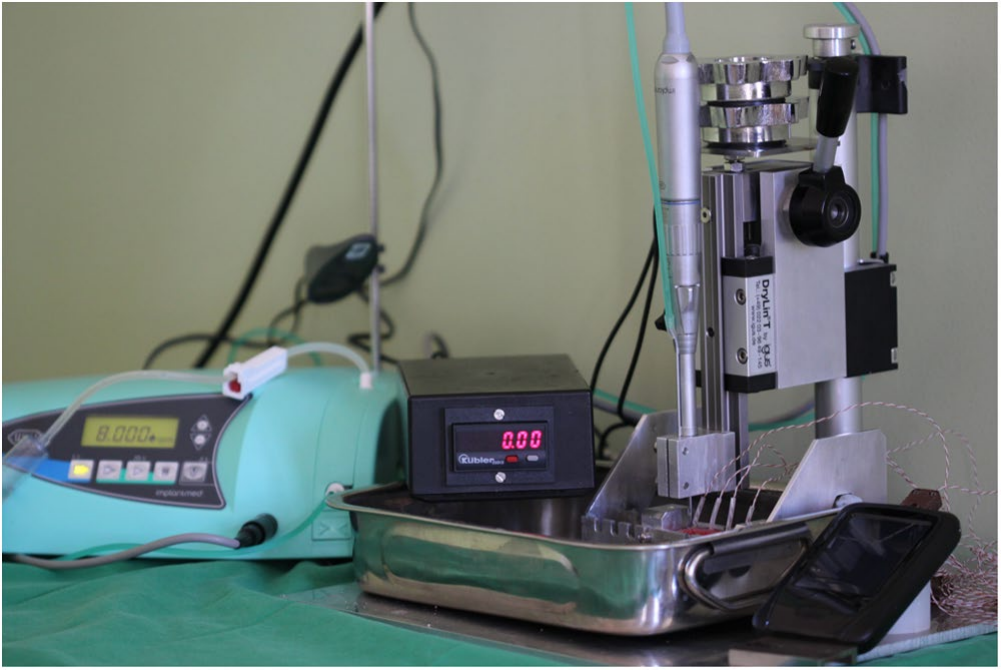
The testing device was completed with a physiodispenser and a surgical straight handpiece. Drilling time was measured by the device automatically (see in the center), while temperatures were measured with thermocouple probes connected to a registration unit (see at lower right corner).

The tested bone removal in our study was an average and everyday bone preparation method in oral surgery^14,15^. Tungsten carbide round drills (HM141A, Hager & Meisinger GmbH, Neuss, Germany) with a diameter of 3.1 mm were used at 8000 revolutions per minute (rpm) speed, with 6 N of axial loading and 60 mL/min physiologic salt irrigation volume. A special testing device was able to standardize drilling parameters (speed, axial load), and a perpendicular, 5 mm in depth cavity drilling^14^. The device cooperated with a physiodispenser unit (Implantmed SI-915, W&H, Bürmoos, Austria) and a surgical straight handpiece (SL-11, W&H) [Fig. 1].

In every of the below detailed groups, 24 drillings were performed, i.e. altogether 24 × 9 = 216 test cavity preparations. All drillings were performed by the same operator (first author), who had remarkable experiences with the drilling tower illustrated in Fig. 1. After twelve drillings, the drill was disposed and a new drill was applied, to disclose the unwanted effect of wearing.

### Bone or bone simulating specimen

The experimental groups were defined according to the type of investigated bones or bone simulation materials: *1)* fresh pig rib; *2)* fresh bovine rib; *3)* 20 PCF (pounds per cubic feet = lb/ft^3^) dens polyurethane (PU) block with 3 mm (50PCF) cortical layer (No. 1522–440, Sawbones Europe AB, Malmö, Sweden); *4)* 20PCF PU without cortical layer (i.e. cancellous block) (Sawbones Europe AB, Malmö, Sweden); *5)* 30 PCF PU with 2 mm (40PCF) cortical layer (30PCF-CP2, Nacional Ossos, Sao Paolo, Brasil); *6)* 30 PCF PU with 1 mm (40PCF) cortical layer (30PCF-CP1, Nacional Ossos, Sao Paolo, Brasil); *7)* 30PCF PU without cortical (Nacional Ossos, Sao Paolo, Brasil); *8)* commercially available poly-methyl-methacrylate (PMMA) (Acrycast, Acrylux Ltd., Tiverton, Devon, UK). Last, but not least in the 9^th^ group, fresh human cadaver ribs were investigated. The specimens were obtained from the Pathology Institute -with their agreement-, University of Pécs, Pécs, Hungary after the Regional Institutional Ethics Committee of University of Pécs approved the research plan and allowed the removal and management of human cadaver bone specimens (7261/PTE/2018). All experiments were performed in accordance with the relevant guidelines and regulations. The mean cortical thickness of the pig ribs was 2.2 mm (±0.09 mm), while in case of the bovine ribs it was 2.3 mm (±0.13 mm). Cortical thickness of human ribs was found to 1.9 mm (±0.15 mm). The cortical thicknesses were measured with a digital caliper, minimum of two measurements were on each bone specimens.

### Set-up for temperature measurements

Experiments were performed in an air conditioned room, at standardized room temperature of 24 °C. Temperature measurements and heat registrations were performed by 0.5 mm in diameter Cu/CuNi thermocouple probes (K type, TC Direct, Budapest, Hungary) and an attached registration device (EL-EnviroPad-TC, Lascar Electronics Ltd., Salisbury, UK) with 1 measurement per second frequency and 0.1 °C resolution.

**Figure 2:**
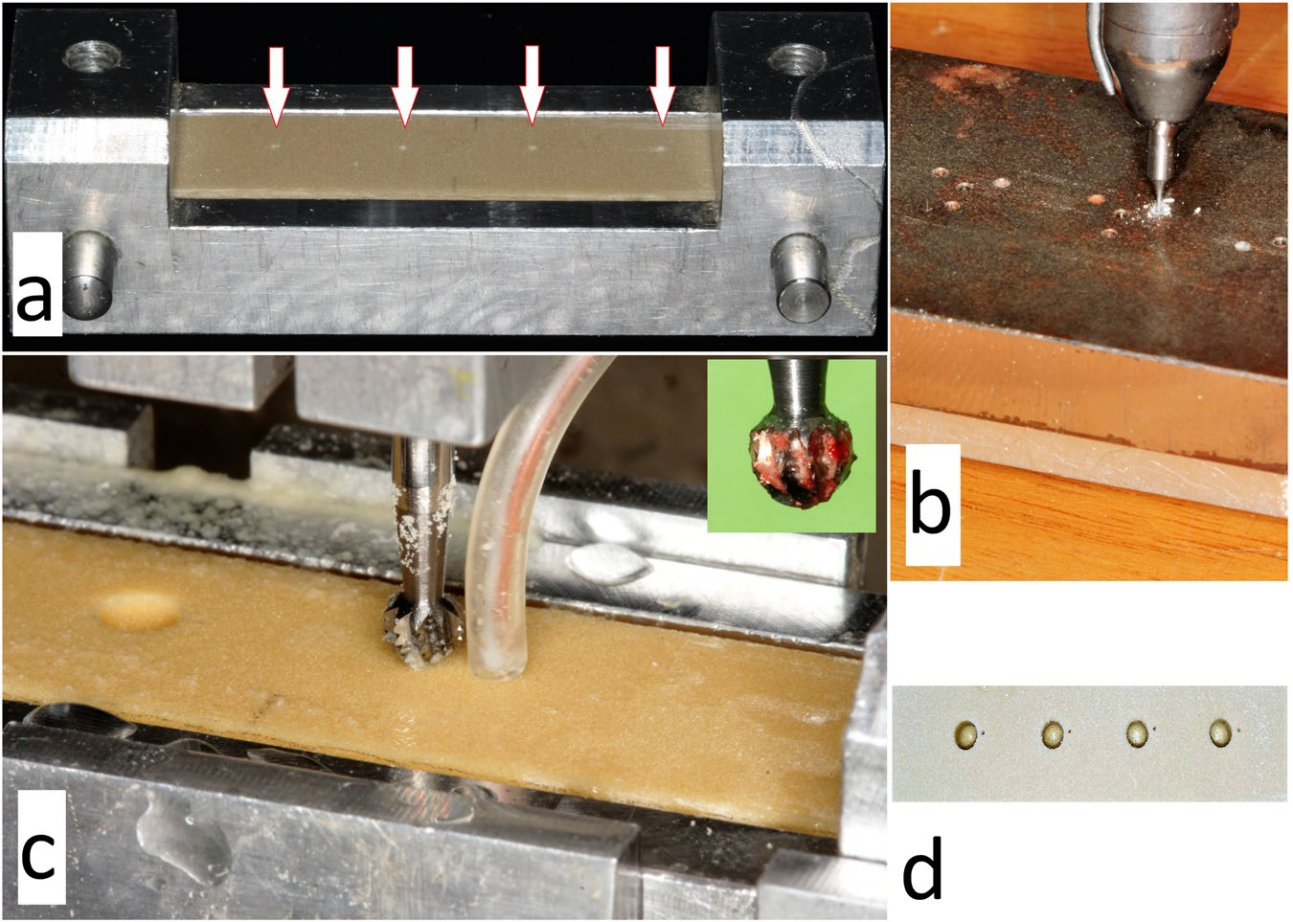
(**a**,**b**) With the help of a metal template the correct localizations and depths of the thermocouple probes were standardized. Arrows indicate thermocouple holes in a polyurethane sample. (**c**) The bone fixating box in one of the predetermined drilling places of the testing device. Note, that polyurethane caused less chip-clogging (caused rather dusting), than e.g. human bone did (see upper right green box in (**c**). (**d**) After drilling, the block proved the correct positions of test cavities and thermocouple holes.

A metal template determined the places of the thermocouple probes in a standard, 1 mm distance from the test cavities, simultaneously allowing placements into the same depth of 5 mm, under the bone surface (Fig. 2). The cavity of the probes was drilled with a 0.5 mm in diameter fissure drill (203 RF, Hager & Meisinger GmbH) (Fig. 2a,b). Probes were isolated with rubber tubes [prepared from 22-G wing needles, B. Braun Melsungen AG, Melsungen, Germany], to disclose the disturbing effect of the cooling liquid (Fig. 2c).

### Statistical analysis

The statistical analyses were performed with SPSS v. 22.0 (SPSS, Chicago, IL). The Kolmogorov-Smirnov test was applied to test the normality of the distribution of the data. The changes in temperatures and drilling times were compared between the tested experimental bone model groups with one-way ANOVA, followed by the Tukey’s HSD post hoc test. P values below 0.05 were considered significant.

### Ethical approval

The Regional Ethics Committee of University of Pécs approved the study (7621/PTE/2018). Animals were not sacrificed for the experiment; bone samples were bought from the butcher.

## Results

**Figure 3:**
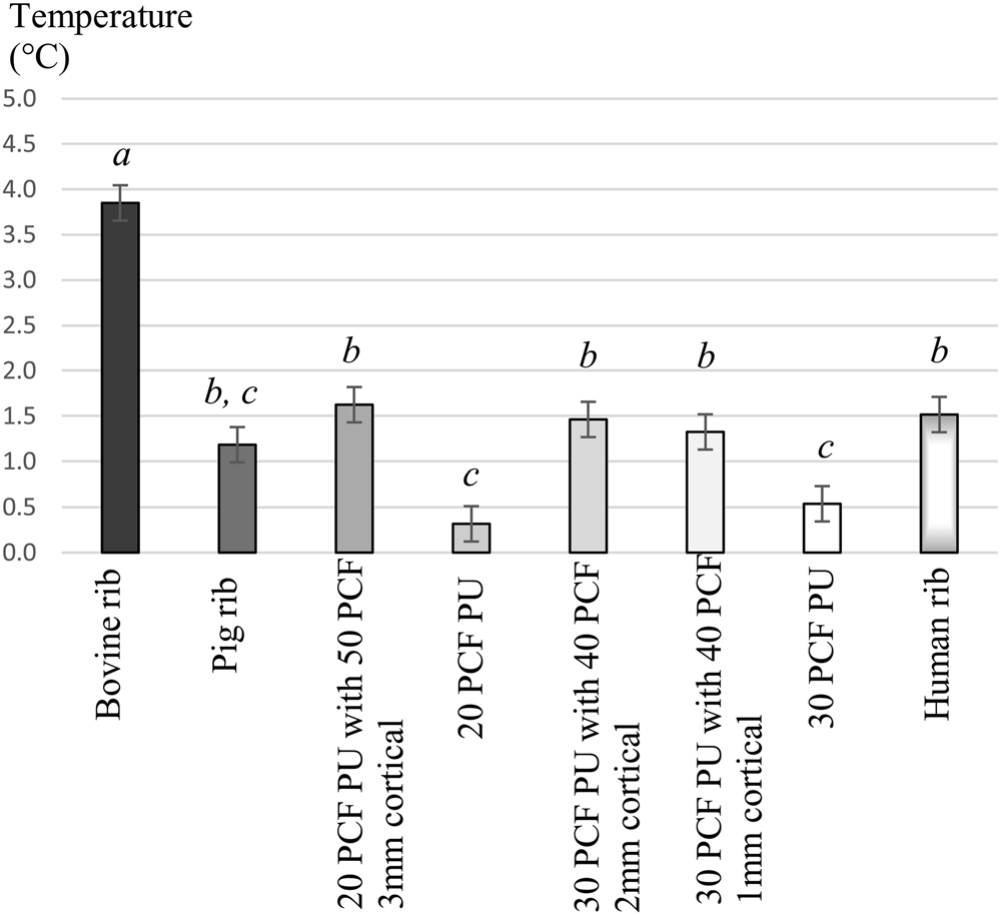
The mean temperature increases and standard deviations during drilling in the tested bone simulation models. Different letters indicate statistically significant differences (1-way-ANOVA, followed by Tukey’s HSD post hoc test). Abbreviations: PCF, pounds per cubic feet; PU, polyurethane.

Mean temperature increases of the drillings (see Fig. 3. and Table 1) were significantly the highest in bovine ribs (p < 0.001). No other investigated model was able to reproduce these temperatures. Temperature increases in human ribs were comparable entirely with polyurethane blocks with cortical layers, or the pig rib (Table 2). 20 PCF PU (p < 0.001) and 30 PCF PU (p < 0.001) showed significantly less heat productions, than human bone. In addition, heat increases in the cancellous PU models were significantly lower, than in PU blocks with cortical layers. Regarding average temperature increases, the 20 PCF and 30 PCF cancellous blocks functioned similarly (p = 0.968).

**Table 1:** The heat productions and drilling times in the different bone models.

The tested bone or bone simulation model	Mean temperature increases (°C)	SD	Mean drilling times (s)	SD
Human rib	1.52	0.19	1.11	0.14
Bovine rib	3.85	1.07	5.08	0.54
Pig rib	1.18	0.28	2.52	1.16
20 PCF PU with 50 PCF 3 mm cortical	1.63	0.32	1.09	0.05
20 PCF PU	0.32	0.12	0.47	0.04
30 PCF PU with 40 PCF 2 mm cortical	1.46	0.40	1.68	0.09
30 PCF PU with 40 PCF 1 mm cortical	1.33	0.24	1.47	0.16
30 PCF PU	0.54	0.11	0.77	0.06

Abbreviations: PCF, pounds per cubic feet; PU, polyurethane; SD, standard deviations.

**Table 2:** Comparisons of the human rib to bone simulation models in this study.

Comparison of investigated drills			Differences of heat productions				Differences of preparation times			
			Differences of the means (°C)	95% CI		P value*	Differences of the means (s)	95% CI		P value*
				Lower	Upper			Lower	Upper	
Human rib	vs.	Bovine rib	−2.34	−2.93	−1.74	**<0**.**001**	−3.97	−4.59	−3.33	**<0**.**001**
		Pig rib	0.34	−0.26	0.93	0.66	−1.41	−2.07	−0.75	**<0**.**001**
		20 PCF PU with 50 PCF 3 mm cortical	−0.11	−0.70	0.49	0.999	0.02	−0.61	0.65	1.000
		20 PCF PU	1.20	0.60	1.79	**<0**.**001**	0.65	0.02	1.28	**0**.**041**
		30 PCF PU with 40 PCF 2 mm cortical	0.05	−0.61	0.72	1.000	−1.93	−2.61	−1.24	**<0**.**001**
		30 PCF PU with 40 PCF 1 mm cortical	0.19	−0.48	0.86	0.986	−1.64	−2.32	−0.96	**<0**.**001**
		30 PCF PU	0.98	0.31	1.65	**<0**.**001**	0.35	−0.36	1.05	0.788

Abbreviations: PCF, pounds per cubic feet; PU, polyurethane; 95% CI, confidence interval.

*Tukey HSD post hoc test.

**Figure 4:**
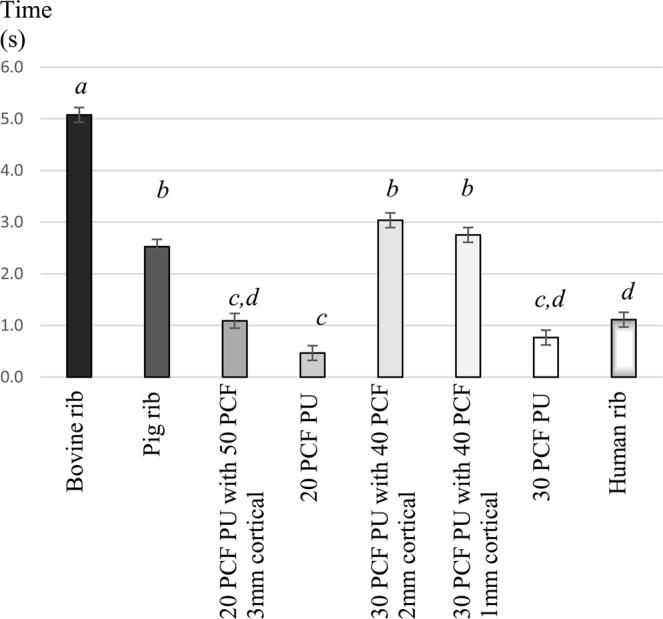
The mean drilling times and standard deviations in the tested bone simulation models. Different letters indicate statistically significant differences (1-way-ANOVA, followed by Tukey’s HSD post hoc test). Abbreviations: PCF, pounds per cubic feet; PU, polyurethane.

Preparation times showed (*see* Fig. 4 and Table 2), that the duration of drilling in bovine rib was significantly the longest (p < 0.001). The pig rib was comparable to Nacional Ossos PU blocks, laminated by 2 or 1 mm cortical layers (p = 0.335 and p = 0.973). Interestingly, the Sawbone PU block with the 3 mm cortical layer was prepared significantly faster, than the pig rib [p < 0.001]. Drilling times in the human rib were comparable to the Sawbone PU block with the 3 mm cortical layer and to the cancellous 30 PCF polyurethane block.

**Figure 5:**
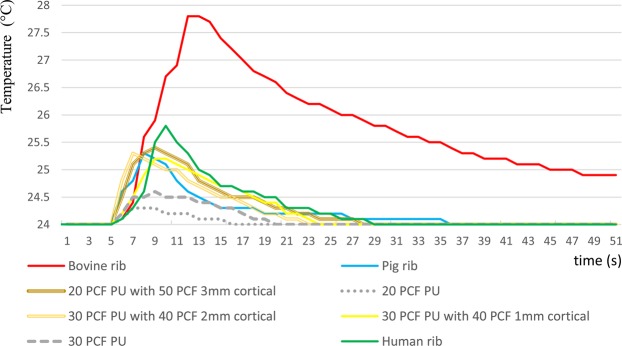
Simultaneous demonstration of eight randomly chosen temperature registration curves of the different bone simulation materials. The polyurethane blocks with cortical layers show similarities with human and pig ribs, however, the temperature characteristics of the bovine rib is very different, including higher maximum values and a more elongated and moderate decrease. Abbreviations: PCF, pounds per cubic feet; PU, polyurethane.

Comparisons of the human bone to the animal bones and PU blocks are represented in Table 2, while eight randomly chosen graphs of drilling temperatures of the study groups are represented in Fig. 5.

## Discussion

Probably the use of drills or other rotating instruments is still the most frequent way of bone removal in the field of oral surgery. Surgeons’ aims are to reduce operation time and patients’ discomfort and to keep thermal parameters acceptable, however an excessive irrigation may disturb the optimal visualization^14^. In contrast, inadequate irrigation or suboptimal drilling conditions may disturb bone regeneration processes and compromise wound healing^6^. The surgeon should apply optimal drilling parameters (revolutions, axial feed, method of irrigation etc.) and chose the correct drill specification (drill diameter and design, drill material, drill wear etc.) to regulate intraoperative temperatures^4,11^.

Intraosseous heat depends on the quality of bone, such as bone density or thickness of the cortical layer^6,16^. Unfortunately, the use of fresh human cadaver bone is limited in the literature, while an optimal, or universally applied or accepted bone substitution model does not exist. Several investigations use an *in vitro* design, examining different animal or synthetic materials to simulate human bone. Animal bones originate usually from pig or bovine - including ribs, mandible, femur, iliac crest-, rarely from sheep. Artificial materials include polyurethane (PU), poly-methyl-methacrylate (PMMA) or hydroxyl-apatite and in one study resin and hardwood was used. Basically, the *in vitro* design has several drawbacks and limitations. There is no blood circulation in the bone or in the model, body temperature can be set and held with difficulties and the parallelism with human *in vivo* characteristics depends highly from the chosen *in vitro* model. While animal bone samples originate from living creatures, which should be a benefit, features change from one individual to the other, exhibiting a variable structure. Since *ex vivo* bone specimens consist of organic and inorganic materials forming a complex anisotropic tissue, it is difficult to investigate the thermal properties. To avoid such variabilities, synthetic polyurethane foam blocks with different densities were introduced^1,2^. This engineering material provides a uniform testing model, showing consistent properties and less susceptible to failures, which require less repetitions^1,2^. Furthermore, the handling and storage of synthetic materials may be much easier.

In case of animal bone models, regarding composition (ash, hydroxyproline, and extractable proteins, IGF-I), density (BMD) or mechanical properties (e.g. fracture stress) different species seems more optimal to simulate human bones than others^17^. Considering before mentioned parameters together, the dog’s bone characteristics seems optimal, while rat’s parameters seems the least, according to Aerssen *et al*.^17^. In *in vitro* investigations, the usage of bovine and porcine or synthetic bones are dominant.

Bone mineral density [BMD] determines drilling temperatures. With the increase of BMD, the hardness of the bone and intraosseous heat increases simultaneously^16,18^. The BMD changes with age, with intraoral bone localization and from patient to patient^18^. The average human BMD was found to be 111.1 lb/ft^3^ (=PCF) (range: 61.2–168.6), the pig BMD was 232.9 lb/ft^3^ (range: 217.2–275.3) and the bovine’s BMD was 280.3 lb/ft^3^ (range: 213.5–334.6) in Aerssen *et al*.’s study^17^. Supposedly, this difference in BMD values might be represented in the significant differences between bovine and pig ribs or between bovine and human ribs in the current study. Regarding thermal characteristics, considering thermal conductivity and specific heat capacity, interesting differences and similarities are seen between human, animal and synthetic bones (Table 3). Data show several times an overlap with humans, but the correct specimen selection might be frequently random and accidental. According to Zdero *et al*. (2016) the 60% of heat is dissipated into bone chips during drilling, while 40% is absorbed by the host bone, so when e.g. a PU synthetic material forms different chips (PU forms rather a kind of powder in our opinion, see Fig. 2c) because of the missing organic compounds, it can hardly mimic the human bone perfectly^19^. When a synthetic material do not form such adhering ‘biological material’ characteristic bone chips, the possibility of chip-clogging (which is a significant temperature increasing factor) of the flutes is reduced^20^. That may partially answer, that temperature increases are not only influenced by similar densities or thermal parameters. Additionally, as Davidson and James concluded, heat capacity (i.e. specific heat) is more important parameter of the material as heat conductivity^21^. While changing the thermal conductivity had a negligible effect on intraosseous temperature, a 10% increase in heat capacity resulted in a 5% drop in temperatures^21^.

**Table 3:** The comparison of the thermal properties of usually applied bone simulating materials.

Mechanical property		Human bone^a–c^	Human Cortical bone^d^	Human Cancellous bone (range)^d^	Polyurethane^e,f^		PMMA^b,g^	Arteficial bovine bone^h,i^	Bovine bone^a,j,k,l^	Porcine bone^a,c^
Density	kg/m^3^	1780–2200	1640	160–640	1700	641	1190–1400	600–1800	4490	2010–3730
	lb/ft^3^ (=PCF)	111–137	102	10–40	106	40	74–87	37.5–112	280	125–233
Specific heat	J/kg °C	1150–1300	1640	1477–1573	1250	1477	1400–1470	not known	~1600	1330
Thermal conductivity	W/m K	0.1–0.35	0.452	0.05–0.087	0.47	0.082	0.15–0.4	0.3–0.4	0.54 (cancellous = 0.3)	0.17

^a^Aerssens *et al*.^17^.

^b^Pandey & Panda^33^.

^c^Hou *et al*.^34^.

^d^Data originate from a finite element analysis model. Chen *et al*.^35^.

^e^Sellani *et al*.^36^.

^f^Data originate from the published technical datasheet of Sawbones^37^.

^g^Data originate from the published technical datasheet of Acrycast^38^.

^h^Strbac *et al*.^13^.

^i^Data originate from the published technical datasheet of Bonesim^39^.

^j^Chen *et al*.^40^.

^k^Davidson & James^41^.

^l^Feldmann *et al*.^42^.

Another interesting difference was found in mammalians’ bone histological structure, i.e. the interspecies differences of Haversian canal diameters, however, its influence on the heat dissipation is obscure. While human Haversian canals’ diameter were found to ~100 µm, that value was in pig and in bovine only ~35 µm^22^.

According to manufacturers’ intentions, the wide variety of densities - i.e. the PCF scale usually from 10 to 50 of PU blocks- should cover the broad spectrum of human bone characteristics. The selected polyurethane blocks in the current study, i.e. from 20 to 50 PCF, with (1–3 mm) or without cortical layers, were selected intentionally, to investigate the simulating potentials of the PU material. The 20 and 30 PCF PU with corticals of 40 and 50 PCF are offered in the literature usually to simulate the typical quality oral bone (D1 and D2 densities according to the classification of Misch)^5,6^. According to the literature, the density of the PU reflects to the human bone densities as follows: D1 = 0.48–0.64 g/cm^3^ = 30–40 PCF; D2 = 0.32 g/cm^3^ = 20 PCF^5,6^. According to our results, PU blocks with cortical layers showed similarities with human and pig ribs, but bovine’s rib showed significantly different results (Figs 3–5). Also, an interesting difference was seen in the drilling times between the two manufacturers who produced PU cortical blocks (Sawbone vs Nacional Ossos). Although Sawbone blocks had thicker cortical (3 mm vs 1 mm and 2 mm) and a higher density of the cortical (50 PCF vs 40 PCF) they needed significantly shorter drilling times. Further model studies need to be conducted to explain that phenomena. Fernandes *et al*. (2016) investigated four PUs with different densities^2^. Cortical was simulated by 50 PCF and 5 PCF blocks and cancellous bone by 20 PCF and 7.5 PCF blocks. It was clear, that temperatures were strongly correlated with densities, but direct comparison with that study is difficult because of the very different drill specifications and drilling parameters, moreover only the drilling in 50 PCF material was cooled with air stream, which is unusual in oral surgery. While *mathematical* (describes usually an overall cutting force) and *numerical* (simulates both forces and material responses) modeling is straightforward, and finite element (FE) analysis can be considered as a solution to estimate the parameters of drilling in order to prevent the problem of osteonecrosis^23,24^, it was interesting to see, that between numerical and experimental temperature increases there was a difference of up to ±2.0 °C, depending on the density of the PU blocks. In a study of Tu *et al*. (2013) the peak temperature difference from the proposed FE model and the experiment was no more than 3 °C^25^. As Mediouni *et al*. (2019), recently stated, “software tools do not replace experimental testing”, but “they provide a valuable and rapidly evolving option”^24^.

Unfortunately, direct comparisons of bone removals in different studies is very difficult, because investigations usually use different bone models, drilling parameters, drill designs or materials. Furthermore, studies using similar bone removals in different bone simulation materials is infrequent^4^. For instance, in two investigations, very similar implant drills were tested (stainless steel 4.2 mm and 4.3 mm in diameter) with similar drilling parameters (20 N axial loading, 1500 rpm)^8,26^ and with similar method of temperature measurements (thermocouple probes). An important difference was, however, the bone simulation material, i.e. bovine femur vs. synthetic polyurethane blocks. The first found an average of 32.1 °C, while the second only an average of 22.2 °C of temperature elevations during implant bed drilling. That was a 30% difference in results. Our results can confirm such a high difference (bovine: 3.85 °C vs PU: 1.63 °C, i.e. ~40% difference), although earlier mentioned researches investigated implant bed drilling with ~12–13 mm deep cavities, while our test cavities were only 5 mm deep. Interestingly, a recent investigation showed, that temperatures during drilling were significantly higher in bovine femurs, than in human cadaveric tibiae^27^. The proportion of temperature increases in human and bovine samples were around 1: 2.65 in the previously mentioned study, while in ours this was very similar, 1: 2.53.

In contrast, in two other investigations both researches investigated similar piezoelectric preparations, but in the first it was performed in bovine ribs, in the second in pig mandibles, and they found very similar average temperature elevations (~3.3 °C vs. ~3.5 °C)^15,28^.

It was clear during our experiments, that not only the storage, but the customization of PU blocks into the bone fixation box was much simpler than in case of animal or human bones. Storage of fresh bones is limited in time without freezing, while frozen bone after unfreeze might change its’ structure and physical properties. Moreover, in case of human bone, several ethical and infection control issues should be managed.

A frequently mentioned drawback of *in vitro* bone simulations is the missing blood circulation, as circulating blood may help to dissipate heat. According to several authors, however, it has less significance on temperature accumulations^11,29,30^. Another noted drawback is the missing physiologic body temperature. Several attempts are seen in researches to simulate ~37 °C body temperature. In some of them, the bone is heated to 37 °C before experiments, in others the room is heated and kept on 37 °C or experiments use a sinking bath [container with preheated physiologic salt], with or without a thermostat. Some opinions, however, suggest that bone sample in a sinking bath may dissipate much less heat, than it would happen *in vivo*, furthermore thermocouple probes may be disturbed by the fluid of the bath^4^. According to Lee *et al*., the insistence to the real 37 °C, as the initial temperature of experiments, can not be proven^31,32^. They showed evidence in two of their researches, that intraosseal temperature changes (ΔT) were similar, when initial temperatures were 26 °C or 37 °C.

**Figure 6:**
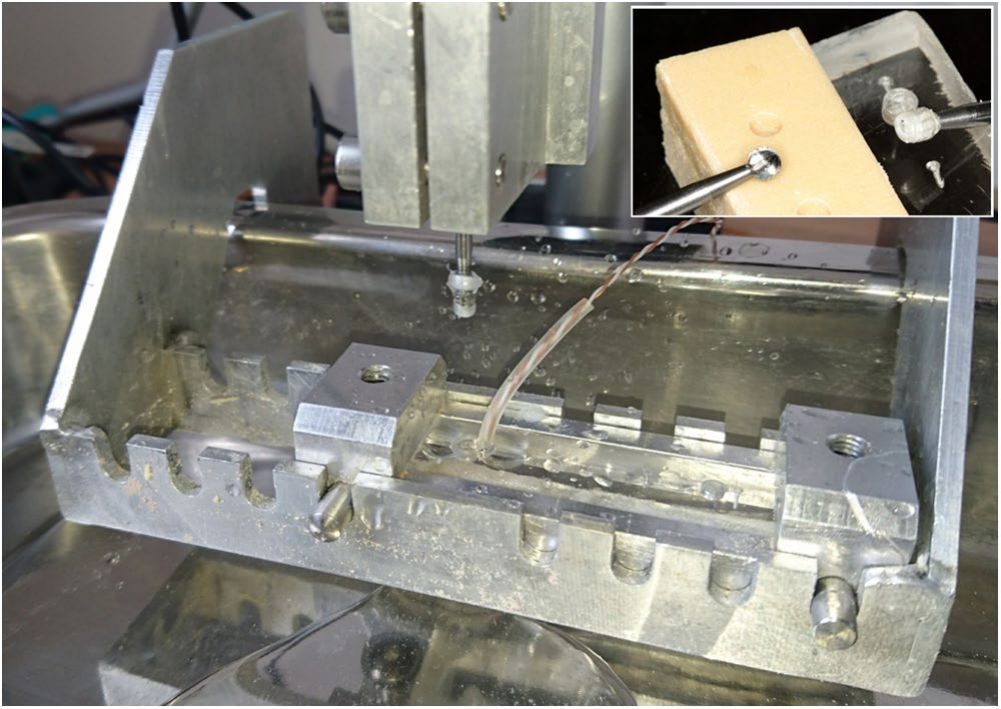
Drilling the poly-methyl-methacrylate (PMMA) with the investigated bone removal parameters was not successful. When axial loading was increased, melting of the PMMA occurred, which ruined the drill. In the right upper corner, the applied drills can be seen after drilling the polyurethane versus the PMMA.

Although, PMMA occurs in more investigations as bone simulation model, according to our experiences it can not be compared with the pig or bovine ribs or the PU. When the places of the thermocouple probes were drilled, we experienced much higher resistance of the material. During drilling, at 8000 rpm and 6 N axial loading, the drills were unable to penetrate into the material. After then, the axial pressure was elevated. At approximately 38–40 N pressure, the drills were able to drill the PMMA, but they needed more then 23–26 seconds to reach the 5 mm depth. During this period, PMMA melted and surrounded, coated the drills (Fig. 6). The removal of the acrylate from the drill was very time consuming. Probes measured an average of 18 °C heat increase during drilling.

Considering the very different drilling and heat increasing conditions of PMMA, authors did not involve these data in the statistical analysis. In contrast, according to Pandey and Panda’s orthopedic simulations^33^, PMMA was prepared with 6–8–10 mm in diameter spiral drills at 2500 rpm, with a feeding rate of 35–45 mm/min successfully, so it can be supposed, that PMMA preparation is possible, however, not for every bone removal simulations. In fact, it is a frequently experienced difference, that orthopedic and trauma related drillings use significantly higher axial pressures than oral and maxillofacial surgery related maneuvers (80–200 N vs. 3–25 N)^3^.

This experimental study had some limitations. With the current drilling conditions (6 N and 8000 rpm), every of the investigated bone model resulted temperatures, which remained significantly under the well-known threshold temperatures of bone necrosis (<10 °C), of peripheral nerve damage (<5–8 °C) or under the threshold of periodontal fibroblast damage (<6 °C)^3^. In case of different drilling conditions e.g. in case of higher axial load or higher revolutions, or when using worn drills or less irrigation, however, it could be possible, that some of the bone specimens would show values below (e.g. pig rib), while other (e.g. bovine rib) significantly higher values than the threshold level. Moreover, the differences of temperature increase between observed bone models might be inconsistent in different drilling conditions, so further studies should investigate inter-model differences with different drilling parameters as well. On the other hand, this study investigated round shaped tungsten carbide drills, while several researches investigate cylindrical twist drills (mini-implant pre-drills, implant bed drills) with higher drilling depths. Another limitation is, that living bone may have different heat dissipation character, so *in vitro* results should be accepted with criticism.

Within the limitations of the study, following conclusions may be drawn:

1. The bovine rib shows significantly higher temperature elevations and slower preparation times than human bone or any other of the investigated simulation models.
2. The pig rib was comparable to human rib, regarding mean heat increases but the drillings in human ribs were significantly faster.
3. The PMMA was an inadequate model by the investigation of our current drilling parameters.
4. Only the 20 PCF dens polyurethane blocks with 50 PCF dens 3 mm thick cortical laminations were able to mimic human ribs regarding drilling temperatures and drilling times simultaneously in this study.
